# Experimental copper exposure, but not heat stress, leads to elevated intraovarian thyroid hormone levels in three-spined sticklebacks (*Gasterosteus aculeatus*)

**DOI:** 10.1007/s10646-020-02278-1

**Published:** 2020-09-25

**Authors:** Ruuskanen Suvi, Mottola Giovanna, Anttila Katja

**Affiliations:** Department of Biology, University of Turku, Turku, Finland

**Keywords:** Thyroid hormones, Plasticity, Maternal effect, Endocrine disruption, Metal pollution, Heat stress

## Abstract

Climate change and pollution are some of the greatest anthropogenic threats to wild animals. Transgenerational plasticity—when parental exposure to environmental stress leads to changes in offspring phenotype—has been highlighted as a potential mechanism to respond to various environmental and anthropogenic changes across taxa. Transgenerational effects may be mediated via multiple mechanisms, such as transfer of maternal hormones to eggs/foetus. However, sources of variation in hormone transfer are poorly understood in fish, and thus the first step is to characterise whether environmental challenges alter transfer of maternal hormones to eggs. To this end, we explored the population variation and environmental variation (in response to temperature and endocrine disrupting copper) in maternal thyroid hormone (TH), transfer to offspring in a common fish model species, the three-spined stickleback (*Gasterosteus aculeatus*) using multiple approaches: (i) We compared ovarian TH levels among six populations across a wide geographical range in the Baltic Sea, including two populations at high water temperature areas (discharge water areas of nuclear power plants) and we experimentally exposed fish to (ii) environmentally relevant heat stress and (iii) copper for 7 days. We found that populations did not differ in intraovarian TH levels, and short-term heat stress did not influence intraovarian TH levels. However, copper exposure increased both T4 and T3 levels in ovaries. The next step would be to evaluate if such alterations would lead to changes in offspring phenotype.

## Introduction

Climate change and pollution are some of the greatest anthropogenic threats to wild populations. Organisms may react to changes in their environment by showing plastic responses (Habary et al. 2017; Parmesan 2006; Stillman and Armstrong 2015). One highlighted form of plasticity is transgenerational plasticity, i.e. when variation in parental environment leads to changes in offspring phenotype (e.g. Bonduriansky and Day 2009; Donelson et al. 2012; 2018; Galloway and Etterson 2007; Metzger and Schulte 2017; Meylan et al. 2012; Mousseau and Fox 1998; Salinas and Munch 2012; Shama et al. 2014; Shama and Wegner 2014), however the molecular mechanisms are not yet understood. Transgenerational effects may be mediated via multiple mechanisms, such as epigenetic markers, transfer of maternal hormones or RNAs to eggs/foetus (e.g. Adrian-Kalchhauser et al. 2018; Best et al. 2018; Kim et al. 2019; Metzger and Schulte 2017; Meylan et al. 2012; Ruuskanen and Hsu 2018).

In vertebrates, hormones transferred from the mother to eggs and embryos are known to profoundly influence offspring development, physiology, morphology, behaviour and even survival (mammals, Dantzer et al. 2013; fish, McCormick 1999; birds, Ruuskanen 2015; Ruuskanen and Hsu 2018; reptiles, Uller et al. 2007). Thyroid hormones are one class of these hormones (THs, prohormone thyroxine T4, and biologically active tri-iodothyronine, T3), that control and regulate vital biological processes, such as thermogenesis and reproduction, but also growth and metamorphosis (Norris and Carr 2013). Recent studies suggest that the role of THs on early development involves maternally transferred hormones (fish, Brown et al. 2014; birds, Hsu et al. 2017; Hsu et al. 2019; mammals, Patel et al. 2011; reviwed in Ruuskanen et al. 2016a). In fish, maternally derived and early-life THs influence development, such as hatching, growth rates, gene expression patterns, and survival (reviewed in Brown et al. 2014, Power et al. 2001, 2014, Ruuskanen and Hsu 2018).

THs are critical regulators of thermal acclimation in fish, increasing in higher temperatures, (e.g. Little et al. 2013) and plasma THs have been found to fluctuate with varying water temperature (e.g. Arjona et al. 2010; Cyr et al. 1998; Eales 1985). However, THs are subject to endocrine disruption by various chemicals, such as pharmaceuticals, pesticides, PCBs, dioxins, and toxic metals, such as lead (Yu et al. 2013; Matthiessen et al. 2018; Norris and Carr 2006; Carr, Patiño 2012; Guyot et al. 2014). A less studied, but potential endocrine-disrupting chemical (EDC) is copper (Cu), although the results are controversial. Copper exposure has been found to both increase and decrease plasma THs in fish, depending on species and timing of exposure (Eyckmans et al. 2010; Hoseini et al. 2016; Oliveira et al. 2008). If egg TH levels are correlated with plasma TH levels of the mother (Kang and Chang 2004; Raine and Leatherland 2003), then any environmentally-induced variation or thyroid disruption in the maternal circulation may influence offspring development via altering maternally-transferred hormone levels (also called transgenerational/ maternal endocrine disruption, e.g. Ruuskanen et al. 2019; Chen et al. 2017).

Surprisingly, studies characterising the environmental or genetic sources of among-female (or within-female) variation in egg TH levels are rare (Ruuskanen and Hsu 2018), yet, such variation could contribute to variation in offspring phenotype and fitness and help to understand the scope for transgenerational plasticity. The first step, therefore, is to characterise whether environmental challenges affect transfer of maternal hormones to eggs. In birds, egg TH levels vary with food availability (Hsu et al. 2016) and temperature (Ruuskanen et al. 2016c), while T3 (but not T4) also shows heritable variation (Ruuskanen et al. 2016b, Hsu et al. 2019). In fishes, there is indication for variation in egg THs among stocks (rainbow trout, *Oncorhynchus mykiss*, Leatherland et al. 1989), which could reflect either genetic or environmental variation. Interestingly, McComb et al. (2005) reported that the egg T3 and T4 concentration in bonnethead sharks (*Sphyrna tiburo)* from Tampa Bay was consistently higher than from eggs from Florida Bay. The authors suggested that this may be due to higher temperatures in Tampa Bay, and speculated that egg THs might explain the faster growth rates and metabolic rates at this site. In the rare examples on endocrine disruption, maternal experimental exposure to pollutants (lead, polybrominated diphenyl ethers and bisphenol A) resulted in decreases plasma and egg THs in zebrafish (*Danio rerio*) (Chen et al. 2017), with delayed larvae development (Wei et al. 2018). The effects of other pollutants, such as copper, on egg hormone transfer have not been addressed, yet the effects of pollutants on plasma levels (see above) suggest that such effects may be likely. However, no systematic investigation into the sources of variation in egg THs have been conducted in fish (Ruuskanen and Hsu 2018).

In the present study, we explored the causes of environmental and population variation in maternal thyroid hormones in a common fish model, the three-spined stickleback (*Gasterosteus aculeatus*). First, six populations from the Baltic Sea, of which two from discharge water areas of nuclear power plants generating higher water temperatures, were used to study the effect of temperature in maternal TH transfer. It is expected that ovarian TH levels in the populations at the vicinity of the nuclear power plants are altered compared to reference areas also in the common-garden conditions, for example due to selection on metabolism.

Second, stickleback populations from different areas in the Baltic (the Gulf of Finland and Gulf of Bothnia) have been found to be genetically different from each other, and the populations somewhat differ e.g. thermal habitats they encounter (Guo et al. 2015). Thus we may also expect overall differences in THs among the six sampled populations, or between the populations in Gulf of Bothnia and Gulf of Finland due to past selection.

Thirdly, we tested the effects of temperature also experimentally by exposing the fish from each population to a mild temperature treatment, mimicking heat stress. We predict that intraovarian T3 and T4 should be higher in the heat stress treatment compared to controls, due to increasing metabolic rates. Finally, contamination in the Baltic Sea is quite high with metals such as copper (but also zinc, lead, cadmium and mercury) reported in sediments, seawater, and e.g. liver tissues of herring and cod, and they influence e.g. physiology in mussels (Lehtonen et al. 2019; Perttila et al. 1982). Given that copper pollution can disrupt thyroid hormone levels in adult fish, we experimentally tested if maternal metal exposure to an environmentally relevant dose of copper can influence intraovarian hormone levels, i.e. allocation of hormones to eggs.

## Materials and methods

### Study areas, catching and maintenance

**Fig. 1:**
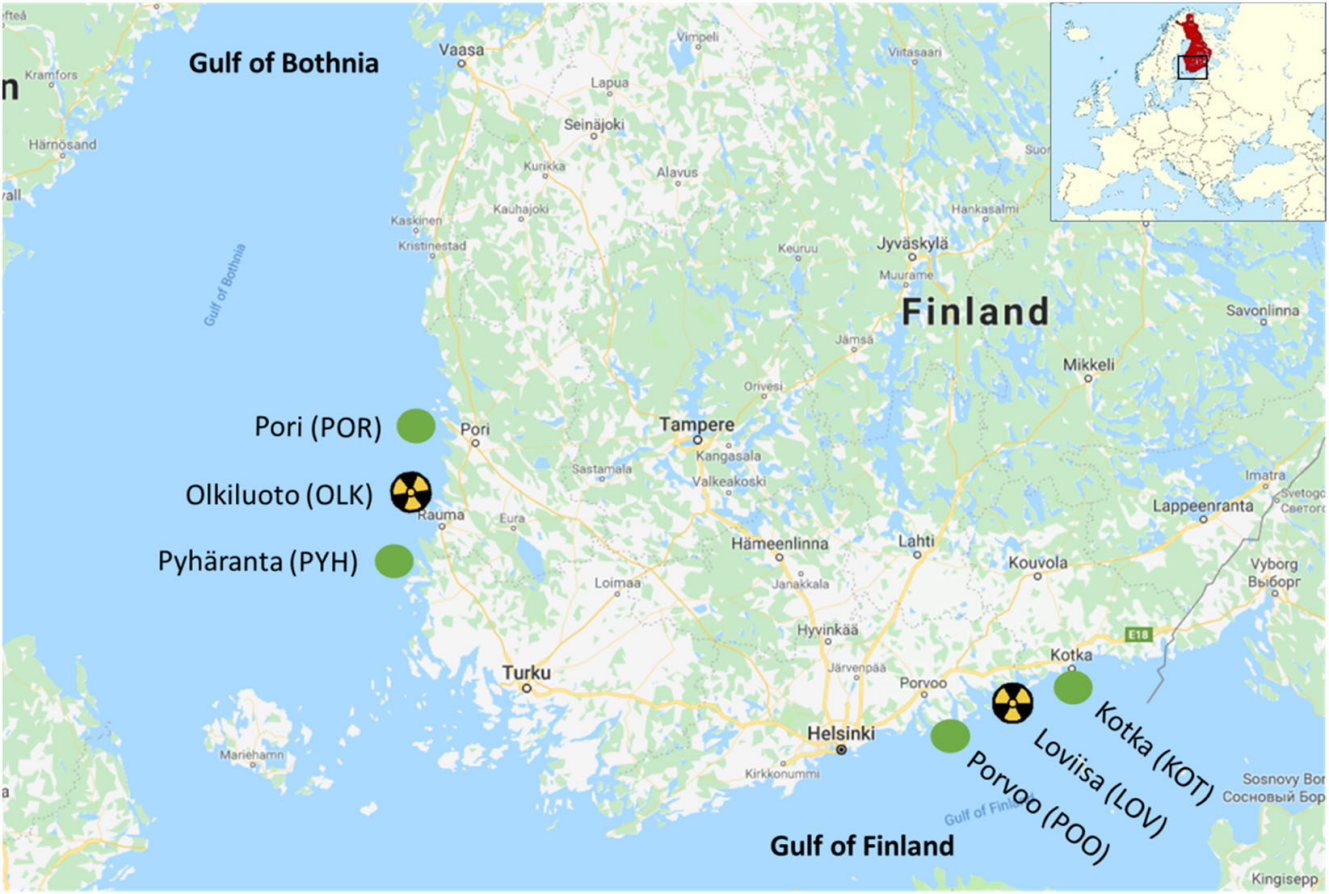
Sampling locations across the Baltic Sea. Yellow-and-black symbols refer to sites with nuclear power plant discharge water and green symbols to reference populations

**Fig. 2:**
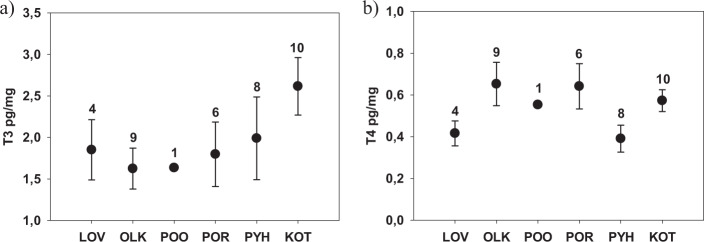
Intraovarian T3 (**a**) and T4 (**b**) concentrations (pg/mg, mean ± SEs) in three-spined sticklebacks from six populations across the Baltic Sea coast (see Fig. 1 for sampling locations). Numbers above the bars refer to sample sizes. POO was excluded from the statistical analysis due to low sample size

The experiments were conducted with wild, adult female three-spined sticklebacks caught from a wide geographical range at six different locations across the Baltic Sea (see Fig. 1). This species was selected as it is an abundant and wide-spread species across the Northern Hemisphere, and an important species in biomonitoring and ecotoxicology as well as in behavioural and ecological studies (e.g. Scholz and Mayer 2008). Three of the locations were in Gulf of Finland and three in Gulf of Bothnia. In both Gulfs one of the locations was in the cooling water discharge area of a nuclear power plant, where discharged water is about 10–12 °C warmer than the surface water and the discharge has continued for ca 50 years (Keskitalo and Ilus 1987), thus these sites can also be viewed as mimicking global warming. The temperature reported in earlier studies was about 3–4 °C higher around the discharge area than further away (Ilus 1983). This higher water temperature at the nuclear power plants could be a potential ongoing selecting agent on thermal physiology, such as metabolism and TH levels. In Gulf of Finland the areas were Loviisa (LOV, Nuclear Power Plant area, N6021.928; E2622.228), Kotka (KOT, reference site, N6026.103; E2652.181) and Porvoo (POO, reference site, N6015.090; E2546.134), see also Fig. 2. In Gulf of Bothnia the areas were Olkiluoto (OLK, Nuclear Power Plant area, N61.2360278; E21.4347222), Pyhäranta (PYH, reference site, N6057.149; E2125.986) and Pori (POR, reference site, N6130.140; E2135.675), see Fig. 2. Temperatures, salinity and pH (±SD) of the locations during catching in May 2018 are presented in Supplementary Table 1. Temperature loggers (three per area, HOBO Water Temp Pro v2 Logger, U22, Onset Computer Corporation, Bourne, MA, USA) were situated directly at the catching locations at the sea bottom (depth 1.5–2 m) and water temperature was recorded 16/5/2018–31/8/2018 from all the locations four times per day.

The adult fish were caught with beach seine net and transferred to University of Turku for rearing. The fish (*N* = 100 per population, mixed sexes, some of the fish were used also in other experiments) were let to acclimate into 180 L tanks at 16 °C for 2 weeks (each population in its own tank). No mortalities were observed during the transfer and the acclimation period in laboratory facilities. Water salinity was 4 ppt (filtered water with 76% NaCl; 20% MgSO_4_; 3.5% CaCl_2_; 0.5% KHCO_3_), pH = 8 and oxygen saturation over 80%. Photoperiod was set to 17L:7D to mimic natural photoperiod. Fish were fed with frozen bloodworms (Delang & Ekman AB/ Akvarieteknik, Sweden) five times per week. The tanks were cleaned and one third of the water was changed once a week in order to avoid accumulation of nitrate, nitrite and ammonia into tanks (tested with Sera GmbH water quality kits, Heinberg, Germany). Upon arrival the fish were treated against nematodes using Nematol (Sera GmbH, Heinberg, Germany) according to the instructions of the manufacturer. In order to reduce any tank effects the fish were tagged intraperitoneally with 1.35 × 7 mm RFID subcutaneous microchips (Loligo^®^ Systems, Viborg, Denmark) under anaesthesia (100 ppm MS-222 in four ppt brackish water buffered with 6 ppm HCO_3_) after 2 weeks’ acclimation period. The populations were mixed into nine tanks (with density of 2 L/fish) and were let to recover for 3 weeks before further testing.

### Experimental treatments

After the recovery period, the fish were exposed to three different environmental conditions in their rearing tanks: control (CTRL7D), sublethal level of copper (Cu 7D) or heat stress (HS 7D) for 1 week. Fish from each sampling location were distributed equally into each replicate tank of all three treatments. Fish were sampled before and after the exposures (see below). The sub-lethal copper exposure (CU 7D) was conducted for a total number of 127 fish in three replicate tanks with mixed populations in each tank. In order to reduce the handling stress which might lead to rise in metabolic rate and thyroid hormone levels (Laidley and Leatherland 1988) the fish (20 females) for thyroid hormone level analyses were taken from one of the replicate tank for the current study. A total of 120 fish in three replicate control tanks (CTRL 7D) were treated similarly but no copper was added to tanks (25 female fish from one tank were sampled for the current study). Water was not changed during the experimental period. For exposure, Cu^2+^ was added manually as copper (II) sulphate pentahydrate solution (nominal: 100 µg/L of CuSO_4_·5H_2_O, Merck, Darmstadt, Germany) to the experimental tanks. This concentration of copper represents environmentally relevant concentrations encountered in polluted waters and was considered sublethal (Sanchez et al. 2005; Gravenmier et al. 2005; Vieira et al. 2009). Water samples were taken both from exposure and control tanks (i) 2 h and (ii) 1 week after the release of copper in order to measure the copper concentration during 1 week of exposure (no water changes were done during the exposure). Fifty millilitres of water were sampled in polypropylene Falcon tubes from tanks. In order to keep water samples fresh prior to analyses, 1 ml of concentrated HNO_3_/100 ml was added to the samples and samples were kept at 4 °C before analyses at SYNLAB Analytics & Services Finland Oy (Karkkila, Finland). The copper levels were measured by using inductively coupled plasma mass spectrometry (ICP-MS) (ThermoFisher Scientific, MA, USA). In the copper exposure treatment, the measured concentrations of copper were 91 (in the tank from which fish were sampled for this study), 101, 91 and 22 µg/L after 2 h and 35 (this study), 48, 37 and 18 µg/L after 1 week in the three exposure and one control tank, respectively. The exposure concentration was relatively high but within environmentally relevant concentration range and has not been shown to cause mortality in sticklebacks in previous studies (Sanchez et al. 2005). According to chemical water analyses all the fish in current study were exposed to low concentration of the copper since they were brought to laboratory facilities, due to the technical purity of salts for producing brackish water. One needs to note that copper is an essential metal, needed for e.g. enzyme cofactors (Festa and Thiele 2011).

A third group of fish (*N* = 125 in total in three tanks, 20 female fish from one tank were sampled for the current study) was exposed to heat stress (HS 7D) for a week during the same time as the other fish were exposed to copper/control treatment. For simulating an environmental heat stress the water temperature in the experimental tanks (16 °C) was gradually increased by 1 °C every 30 min until reaching 26 °C and kept at this temperature for a week. The warming experimental set-up was done with chiller-heater (Julabo, Model: F32; AC: 230 V 50/60 Hz 12 A, Julabo GmbH, Seelback, Germany) connected to stainless steel coils. Water was not changed during the experimental period.

### Sampling procedure

Before exposing fish to heat stress or copper, 17 female fish were sampled from a control tank (hereafter CRTL). Fish were sacrificed with cranial percussion. Tag code, weight (g), length (cm) were recorded from each fish. For the current study the ovaries were collected and flash frozen in liquid nitrogen. Samples were stored at −80 °C for further molecular analyses. Similar sampling of the fish and ovaries was conducted also seven days after exposure for control (CTRL7D), copper exposure (Cu7D) and heat stress treatment (HS7D) for a total number of 65 female fish. See sample sizes per treatment and population in Supplementary Table 2.

### Population comparisons

In addition to the experimental dataset, we described the differences among the six sampling locations (populations), and the differences among the two Gulfs (assumed to be genetically differentiated), in TH concentrations (Fig. 2). We pooled all individuals in the CRTL and CRTL7D in this dataset to increase the sample size (see sample sizes for each population in Supplementary Table 2). Given that fish were acclimated 5 weeks before sampling, we believe that additional 7 days in the same conditions should not drastically influence their physiology, and thus pooling was justified.

### Thyroid hormone analyses

**Fig. 3:**
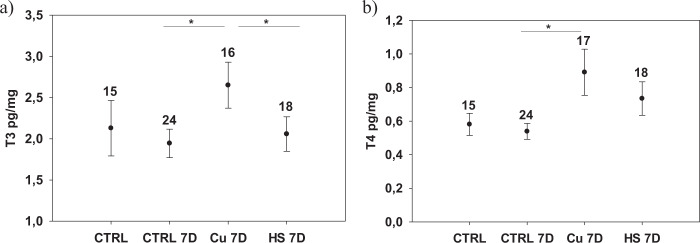
Intraovarian T3 (**a**) and T4 (**b**) concentrations (pg/mg, mean ± SEs) in three-spined sticklebacks experimentally exposed to copper (100 µg/L, CU 7D), warm temperature (10 °C increase, HS 7D) for 7 days and respective controls (CTRL 7D and CTRL – sampled before start of the experiment). Numbers above the bars refer to sample sizes. Stars represent significant differences between treatments at *p* < 0.05. For the statistical analyses the populations were pooled

The whole content (i.e. egg follicles and associated ovarian fluids) was gently squeezed out of the thawed ovaries directly into a microcentrifuge tube. The sample was thereafter weighed (~0.001 g). Samples were analysed for T3 and T4 using LCMS/MS at the facilities of Turku Center for Biotechnology. T4 and T3 were extracted from the sample following previously published methods (De Escobar et al. 1985, Ruuskanen et al. 2019). In short, samples were homogenised in methanol using a tissue lyser (Qiagen, Retsch GmbH, Haan, Germany). As an internal recovery tracer, a known amount of ^13^C_12_-T4 (Larodan, Sweden) was added to each sample. This allowed us to control for the variation in recovery (i.e. extraction efficiency) for each sample. Next, 400 µl of chloroform was added to sample. After centrifugation (15 min, 1900 g, 4 °C), the supernatant was collected and the pellet was re-extracted in a mixture of chloroform and methanol (2:1). Back-extraction into an aqueous phase (0.05% CaCl_2_) was followed by a re-extraction with a mixture of chloroform:methanol: 0.05% CaCl_2_ (3:49:48) and this phase was further purified in-house on Bio-Rad AG 1-X2 (USA) resin columns. The iodothyronines were eluted with 70% acetic acid, and evaporated under N_2_. Blanks (plain reagents without any sample) were analysed in each extraction batch to detect any contamination. Samples from different populations and treatments were equally distributed across four extraction batches. There was no difference among the batches in hormone concentrations (*F* < 1.0, *p* > 0.05). T3 and T4 were quantified using a nanoflow liquid chromatography-mass spectrometry (nano-LC-MS/MS) method, developed and validated in Ruuskanen and Hsu 2018. Briefly, before the analysis, the dry samples were diluted in ammonium (NH_3_)_._ Internal standards ^13^C_6_-T_3_ and ^13^C_6_-T_4_ (Sigma-Adrich, St.Louis, USA) were added to each sample to identify and quantify the THs. A triple quadrupole mass spectrometer (TSQ Vantage, Thermo Scientific, San Jose, CA) was used to analyse the samples. For the chromatographic separation of hormones, a nanoflow HPLC system Easy-nLC (Thermo Scientific) was applied. On-column quantification limits were 10.6 amol for T4 and 17.9 amol for T3. MS data was acquired automatically using Thermo Xcalibur software (Thermo Fisher Scientific) and analysed using Skyline (MacLean et al. 2010). For the analyses, peak area ratios of sample to internal standard were calculated. TH concentrations are expressed as pg/mg fresh mass. Few samples failed in the extraction, see exact samples sizes in Figs. 2 and 3 and Supplementary Table 2.

### Statistical analysis

All statistical analyses were conducted with SAS Enterprise Guide version 7.1. T4 concentration (pg/mg) was log-transformed to reach normality. We first analysed differences in intraovarian T3 and T4 among long-term heat exposed (i.e. populations in the vicinity of the nuclear power plants that are exposed to warm discharge waters; pooled LOV and OLK, *N* = 13 individuals) and reference populations (pooled POO, POR, KOT, PYH, *N* = 24 individuals) using a linear mixed model, using fish mass as a covariate and population as a random intercept. This comparison was restricted to fish monitored under control conditions treatments (CRTL and CRTL7D). The average concentrations of T3 and T4 were similar between CRTL and CRTL7D; T3: 2.1 (SD 1.3) vs. 1.9 (SD 0.8) pg/mg, and T4: 0.58 (SD 0.25) vs. 0.54 (SD 0.23), respectively, suggesting that such pooling strategy was justified. We then analysed differences in intraovarian T3 and T4 concentration among all six populations again using data only from the control treatments. From POO, we only had 1 individual for this analysis, and thus this population was excluded from the analysis. Population and fish mass were included as fixed effects. Next, we pooled the data for the three populations in each Gulf, and further analysed potential larger scale population differences across the two sites using linear mixed models.

Differences in ovarian T3 and T4 concentration between treatments (CRTL 7D, Cu 7D, HS 7D) were analysed using linear mixed models where treatment and fish body mass were included as fixed effects and population as a random intercept to account for non-independence of fish from the same population. See sample sizes from each population in each treatment group in Supplementary Table 2. Finally, we also repeated the above models for fish size (mm). Post-hoc tests were further used to test pair-wise differences among treatments. Models were reduced by removing non-significant factors (*α* = 0.05). Normality and homoscedasticity of the residuals were visually inspected. Degrees of freedom were estimated with Satterthwaite estimation method. Means and standard errors (SE) are shown in the text and in the figures. Cohen’s d and power was further calculated to estimate effect sizes.

## Results

### Fish size

Fish size did not differ among exposed and reference populations (*F*_1,35_ = 1.89, *p* = 0.18), while there were some differences among populations (*F*_4,32_ = 6.92, *p* = 0.004): fish from KOT were significantly smaller than those from OLK, PYH and POR (see Supplementary Table 3).

### Among-population variation in thyroid hormones

When using data from control treatments only (pooled CRTL and CRTL 7D), and comparing all six populations, we found no strong statistical evidence for differences among populations in intraovarian T3 or T4 concentration (T3: *F*_4,30_ = 0.50, *p* = 0.73, T4: *F*_4, 30_ = 2.38, *p* = 0.08; Fig. 2a, b). Body mass correlated negatively with intraovarian T3 concentration (estimate ± SE: −0.842 ± 0.363, *F*_1,30_ = 5.37, *p* = 0.027), but not with T4 concentration (*F*_1, 30_ = 0.0, *p* = 0.96). We also found no evidence that T4 or T3 from fish from the Gulf of Bothnia and Gulf of Finland would differ (T4 *F*_1,5.9_ = 0.1, *p* = 0.91; T3 *F*_1,36_ = 3.55, *p* = 0.07).

We found no differences in intraovarian T3 or T4 concentration in sites exposed to warm discharge waters (pooled LOV and OLK) compared to reference sites (pooled four reference sites, T3: *F*_1,36_ = 0.88, *p* = 0.18, T4: *F*_1,3.75_ = 0.01, *p* = 0.92; Mean (pg/mg) ± SE: T3 in exposed populations: 1.60 ± 0.19, T3 in reference populations: 2.21 ± 0.24; T4 in exposed populations: 0.57 ± 0.08; T4 in reference populations 0.53 ± 0.04). The effect size (Cohen’s d, calculated from raw data) was also small, 0.27, and with the current sample sizes a power analysis revealed low power (0.15). 213 individuals per group would have been needed to reach the recommended power of 0.8. These large numbers indicate that the biological differences across the exposed and control sites are likely small.

### Effects of experimental copper and heat exposure on thyroid hormones

Fish size did not differ among the treatments (*F*_2,51_ = 0.64, *p* = 0.53). 4% (5 out of 127) fish died in copper treatment, while none died in the control treatment (*N* = 120). After experimental exposure to copper for 7 days, fish from copper exposure group had higher intraovarian T3 and T4 concentrations compared to fish from control treatment (T3: overall test *F*_2,53.1_ = 3.14, *p* = 0.05, post-hoc CTRL7D vs. CU7D *t*_52.3_ = −2.30, *p* = 0.02; T4: *F*_2, 53_ = 2.62, *p* = 0.08, CTRL7D vs. CU7D *t*_53.8_ = −2.26, *p* = 0.027, Fig. 3a, b). A power analysis for the CTRL7D vs. CU7D comparison for T3 yielded a power of 0.57 with the current sample sizes, suggesting that we have moderate power. Also, the effect size (Cohen’s d) was moderate to large, 0.61. To further analyse whether timing of sampling differentially influenced hormone measurements, we additionally ran a model with both CTRL and the other 3 treatment groups. T3 did not differ between CRTL and CU7D (T3 *t*_64.5_ = 0.74, *p* = 0.48) while there was a tendency for higher T4 in CU7D than CTRL (*t*_67_ = −1.87, *p* = 0.06), but no differences between CTRL and HS 7D (*p* > 0.45) for either hormone.

T3 or T4 concentrations of fish from the heat treatment (HS7D did not differ statistically from control treatment (CTRL7D or CTRL post-hoc *p* values > 0.45), while ovarian T3 concentration was lower in heat treatment compared to copper exposed fish (*t*_52_ = 2.52, *p* = 0.045, Fig. 2a). In the CTRL7D vs. HS7D comparison, power for T3 with the current sample size was 0.06, and 930 individuals would have been needed to make this difference statistically significant. The effect size (Cohen’s d) was, of course, also negligible, 0.13. These results suggest the effect of heat was biologically very small. 4.8% of fish (6 out of 125 fish) died during the heat stress treatment.

## Discussion

In contrast to our hypothesis, we found no evidence that intraovarian TH levels of individuals originating from populations close to a long-term heat source (nuclear power plant discharge waters), differ from individuals originating from reference populations. It might be that longer duration of heat exposure and/or larger selective pressure is needed to see genetic changes in variation of THs and related traits. Because in the current study the fish were exposed to high temperatures near nuclear power plants for about 50 years, and stickleback generation time is 2 years, there might not have be long enough time to induce genetic changes.

Furthermore, the six populations sampled across a wide geographical range over the Baltic Sea showed similar intraovarian TH levels after acclimation in captivity. We also found no evidence that fish T3 or T4 levels from Gulf of Finland would differ from those from the Gulf of Bothnia. Given that hormone levels were measured in standardised conditions, the results suggest that populations may not show genetic variation in intraovarian T4 or T3 levels in these populations. The largest genetic differences in Baltic Sea were indeed detected between the Danish and Northern Baltic Sea stickleback populations, and not between the two Gulfs (Guo et al. 2015). However, we cannot rule out that environmental sources of variation (temperature, salinity, food availability) could influence intraovarian THs in the wild and the plastic responses in THs might have led to similar THs levels between populations when reared in common garden conditions. For example, bonnethead sharks showed among-population variation in egg THs (McComb et al. 2005). Yet, our sample size was low in the among-population comparison, and the differences in environmental conditions (see Supplementary Table 1) among the populations were rather small at the time of the sampling, thus the results should be interpreted with extreme caution.

Following our prediction on copper as an EDC and specifically, a thyroid hormone disrupting agent, we found that individuals exposed to environmentally relevant concentrations of copper for seven days showed higher intraovarian T3 and T4 levels than controls. If these changes would influence offspring traits, such transgenerational endocrine disruption may present a new pathway for potentially harmful transgenerational effects in this species. While associations between copper and maternal THs have not been studied to date in fish, previous studies on the associations between copper as an EDC and THs in plasma show complex patterns depending on the duration of the exposure and species, and potentially life-stage, as reported in Eyckmans et al. (2010): In common carp (*Cyprinus carpio*), T3 levels were elevated only after long-term exposure (1 month), while in gibel carp (*Carassius gibelio*) there was a decrease in T3 from 24 h to 1 month of exposure. Both species showed increases in T4 over short-and long-term exposure. In rainbow trout, T4 levels were elevated very fast after copper exposure and remained elevated for 12 h, whereas there was no influence on T3. Copper exposure also increased plasma T4 in the common carp in another experiment (Hoseini et al. 2016). Finally, copper exposure significantly decreased T3 but not T4 in European eels (*Anguilla Anguilla*) (Oliveira et al. 2008), suggesting changes in deionisation from T4 to T3 in tissues. The fish in each treatment group was a mix originating from different populations, which suggests that the effect of copper is rather strong and not dependent on the origin of the fish. However, we need to acknowledge that the fish for the current study were sampled from a single tank per treatment, thus, we cannot fully exclude some tank effect, even though the tanks, fish densities and all the rearing conditions were the same for each tank. Furthermore, we report that there were no statistically significant differences in T3 among individuals sampled at the start of the experiment (CRTL) and after 7 days of copper exposure, suggesting potentially some changes in T3 levels with time. However, such patterns were not found for T4.

The association between plasma and intraovarian/egg hormone levels in fish has not been fully elucidated, but if we assume that there is some correlation between intraovarian and circulating TH levels (Ayson and Lam 1993; Brown et al. 1988; 2014; Kang and Chang 2004; Raine and Leatherland 2003), our results are in parallel with those of common carp (see above). Interestingly, our hormone measurements of the two forms (T3 higher than T4) were contrasting compared to whole body measurements in the same species (T3 lower than T4, Gardell et al. 2017), and egg measurements on other species (T3 lower than T4, Chen et al. 2017). We can speculate that this may be due to differential deposition of the two forms, or deiodinase function, converting T4 to T3 in the ovaries in these species and sample type. The increased T4 and T3 levels in response to copper exposure reported in this study suggest that T4 biosynthesis or degradation was altered, but also that conversion of T4 to T3 was potentially increased. Increased TH levels could be explained by increased metabolic rates and energy expenditure in response to copper exposure, along with increased oxidative stress (De Boeck et al. 2006; 1997; Sanchez et al. 2005). All in all, this evidence suggests that copper exposure changes plasma and associated ovarian TH levels. The next step would be to evaluate whether such changes lead to changes in offspring development, metabolism and thermotolerance.

In contrast to our predictions, we did not find any differences in TH concentrations between experimental heat treatment and control. In a previous study it has been found that 3 weeks acclimation to high and low temperature changed the muscle TH profile of zebrafish, warm acclimated fish having higher concentrations of T3 and T4 than cold acclimated ones (Little et al. 2013). We can speculate that perhaps the exposure duration (7 days) was not long enough to elicit changes in TH concentrations. However, in their study the warm-acclimated fish were not sensitive to changes in hormone levels, potentially due to lack of change in transporters/receptors, suggesting that with temperature exposure also e.g. TH transporters and receptors need to be evaluated.

We conclude that variation in intraovarian THs was not explained by variation among populations nor short-term heat exposure. Given that parental temperature environment is known to alter offspring phenotype (e.g. Donelson et al. 2012; 2018), further studies are needed to elucidate the molecular mechanism of such transgenerational effects. Both T4 and T3 levels in ovaries were altered in response to moderate copper exposure, and now the next step is to characterise potential functional consequences of altered THs on offspring phenotype, which would allow us to understand the scope for transgenerational endocrine disruption. Furthermore, other environmental factors such as salinity may be responsible for driving variation across populations as it is know that (1) TH levels respond to variation in salinity (e.g. Moreira et al. 2018) and (2) stickleback adaptive divergence from marine to stream environment has been found to involve thyroid hormone signalling (Kitano and Lema 2013; Kitano et al. 2010). By pooling the three populations from Gulf of Bothnia (somewhat higher salinity) and Gulf of Finland (somewhat lower salinity), we could, however, not demonstrate differences among the sites in TH concentrations. Yet the range of salinity differences remained very small in this study, and thus a broader geographical range/range of salinities would be needed to address this question in the future.

## Supplementary information

Supplementary Tables
