# Supplementary material for: Experimental copper exposure, but not heat stress, leads to elevated intraovarian thyroid hormone levels in three-spined sticklebacks (Gasterosteus aculeatus)
Source: Ecotoxicology. 2020 Sep 25;29(9):1431–40. doi: 10.1007/s10646-020-02278-1 (PMC7581574; doi:10.1007/s10646-020-02278-1)
Supplement: Supplementary file 1 — Supplementary Tables [file 10646_2020_2278_MOESM1_ESM.docx]

Supplementary Table 1. The environmental variables (mean±SD) of catching locations across the Baltic Sea. The

| Location | Temperature during catching, ºC | salinity (ppt) | pH | Average ± SD temperature 16/5/–31/8/2018 |
| --- | --- | --- | --- | --- |
| LOV | 19.9 ± 1.6 | 3.2 ± 0.2 | 7.6 | 19.6 ± 4.4 |
| OLK | 20.1 ± 0.1 | 5.3 ± 0.0 | 9.0 | 19.9 ± 3.6 |
| POO | 18.8 ± 0.5 | 4.3 ± 0.0 | 9.0 | 19.1 ± 4.2 |
| POR | 18.9 ± 0.8 | 4.9 ± 0.0 | 9.0 | 19.7 ± 3.4 |
| PYH | 17.6 ± 1.1 | 4.2 ± 0.4 | 9.0 | 19.5 ± 2.5 |
| KOT | 18.1 ± 0.7 | 1.7 ± 0.0 | 9.0 | 17.3 ± 4.9 |

Footnote: Previous studies have reported ca 3-4ºC differences among sites close to nuclear power plants (LOV and OLK) vs sites further away (e.g. Ilus, 1983). Due to unusually warm weather in the sampling year, differences among sites remained small. Yet, we aim to test for effects of long-term (50 years) of warming instead of current local temperatures.

Supplementary Table 2. Final sample sizes used for hormone measurement in each of the four treatment groups by study location (the six study populations). See text and Fig 1 for description of the sampling sites.

|  | **Populations** | | | | | |  |
| --- | --- | --- | --- | --- | --- | --- | --- |
| **Treatment group** | KOT | LOV | OLK | POO | POR | PYH | **Total N /treatment** |
| CTRL | 6 | 1 | 2 |  | 1 | 4 | 15 |
| CTRL 7D | 4 | 3 | 7 | 1 | 5 | 4 | 24 |
| Cu 7D | 4 | 3 | 4 | 2 | 1 | 3 | 17 |
| HS 7D | 6 | 1 | 2 | 2 | 1 | 5 | 18 |
|  |  |  |  |  |  |  |  |
| **Total N /population** | 20 | 8 | 15 | 5 | 8 | 16 | 74 |

Supplementary Table 3. The average lengths (mm, SE) of fish captured from six different locations (populations) across the Baltic Sea. Populations with different letters are statistically significantly different from each other (p <0.05, Tukey post-hoc test). POO was not included in the statistical analyses due to small sample size.

| Location | Fish size, mm  average (SE) |
| --- | --- |
| LOV | 6.4 ± 0.4ab |
| OLK | 6.6 ± 0.3b |
| POO | 6.1 ± 0.7 |
| POR | 6.9 ± 0.3b |
| PYH | 6.5 ± 0.3b |
| KOT | 5.2 ± 0.2a |
